# Optimization of Growth Conditions for Purification and Production of L-Asparaginase by* Spirulina maxima*

**DOI:** 10.1155/2016/1785938

**Published:** 2016-07-25

**Authors:** Hanaa H. Abd El Baky, Gamal S. El Baroty

**Affiliations:** ^1^Plant Biochemistry Department, National Research Centre, P.O. Box 12026, Dokki, Cairo, Egypt; ^2^Biochemistry Department, Faculty of Agriculture, Cairo University, Cairo, Egypt

## Abstract

L-asparaginase (L-AsnA) is widely distributed among microorganisms and has important applications in medicine and in food technology sectors. Therefore, the ability of the production, purification, and characterization of AsnA from* Spirulina maxima* (SM) were tested. SM cultures grown in Zarrouk medium containing different N_2_ (in NaNO_3_ form) concentrations (1.25, 2.50, and 5.0 g/L) for 18 days contained a significant various quantity of dry biomass yields and AsnA enzyme levels. MS L-AsnA activity was found to be directly proportional to the N_2_ concentration. The cultures of SM at large scales (300 L medium, 5 g/L N_2_) showed a high AsnA enzyme activity (898 IU), total protein (405 mg/g), specific enzyme activity (2.21 IU/mg protein), and enzyme yield (51.28 IU/L) compared with those in low N_2_ cultures. The partial purification of crude MS AsnA enzyme achieved by 80% ammonium sulfate AS precipitated and CM-Sephadex C-200 gel filtration led to increases in the purification of enzyme with 5.28 and 10.91 times as great as that in SM crude enzymes. Optimum pH and temperature of purified AsnA for the hydrolyzate were 8.5 and 37 ± 0.2°C, respectively. To the best of our knowledge, this is the first report on L-asparaginase production in* S. maxima*.

## 1. Introduction

Enzymes are proteins that catalyze biochemical reactions. They are more important than vitamins and minerals for general health [1]. Hydrolases constitute a class of enzymes widely distributed in nature from bacteria to higher eukaryotes. L-asparaginase (L-asparagine amidohydrolase EC, 3.5.1.1) is an enzyme of high intensive chemotherapeutic application due to its use in wide variety of cancer therapy mainly in acute lymphoblastic leukemia [2]. It is used for treatment of various diseases such as childhood acute lymphoblastic leukemia, myelomonocytic leukemia, reticulum sarcoma, melanoma sarcoma, non-Hodgkin's lymphoma, pancreatic carcinoma, and bovine lymphoma sarcoma [3, 4]. The enzyme deprives these types of cancer of L-asparagine because it catalyzes the deamination of L-asparagine, an essential amino acid for lymphoblast growth, to L-aspartic acid and ammonia thus shrinks these tumors [5]. Since the tumor cells require the high quantity of asparagine from the diet and from other cells for their rapid proliferation and depend on an external supply of L-asparagine for their growth, therefore, by continuous administering of L-asparaginase, the tumor cells are made to stave off that particular amino acid, which leads to the death of lymphoblasts by apoptosis [6]. These AsnA enzymes are produced by a large number of microorganisms that include bacteria (*Streptomyces gulbargensis*,* Enterobacter cloacae*,* and Serratia marcescens*), fungi, and yeast. All the forms of the enzyme have similar functionality and received important attention [7–11].

Recently, L-asparaginase is used in food technology as a potent mitigating agent for reducing the acrylamide (AA, CH_2_=CH-CO-NH_2_), a potential carcinogen, which is formed from the reaction of L-asparagine (L-AsnA) and reducing sugars contained in foods during heating processes [12]. However, a wide range of foods prepared by heating treatments above 160°C like coffee, bread, fried or roasted potato products, toasted bread, and sweet biscuit contained high amounts of AA (30–5,600 ng/g) [13]. Mohan Kumar et al. [14] have reported that AA had an adverse effect on human health and is proven to be neurotoxic, genotoxic, carcinogenic, and toxic to the reproductive system. Consequently, it is important to the application of AsnA in processed foods to suppress the formation of AA content to minimal levels, which could correspond to a negligible cancer risk in final food products [15].

More recently, production of L-asparaginase from blue-green microalgae is receiving more attention due to its high nutrient contents, low cost of production, and cost-effectiveness, no seasonal variation, high efficient producers, being easily cultured and harvested at large scales, and cheaper and easier extraction, and higher yields and purification of protein and enzymes by simple methods are available [16]. However, few specific reports regarding production of AsnA by blue-green algae are recorded [16]. Thus, there is a need to identify algae species which produce high levels of asparaginase under low cost and simple culture conditions. Considering the importance of asparaginase, this study was carried out to produce optimized and characterized L-asparaginase from SM and determine the purification process and characterization of its properties.

## 2. Material and Methods

### 2.1. Algal Strains

Blue-green alga,* Spirulina maxima*, was cultivated in Plant Biochemistry Department, Algae Unit, National Research Centre, Cairo, Egypt.

### 2.2. Reagents

All reagents and chemicals used in this experiment were of analytical grade and purchased from Sigma-Aldrich Chemicals.

### 2.3. Cultivation of Algae

#### 2.3.1. Preparation of* Spirulina maxima* Inoculums

*S. maxima* was cultivated (during spring season 2014, in National Research Centre, Egypt). It was grown in the modified Zarrouk medium [17] containing the following nutrients (gL^−1^): Na_2_CO_3_, 4.03; NaHCO_3_, 13.61; NaCl, 1.00; K_2_SO_4_, 1.00; NaNO_3_, 2.50; K_2_HPO_4_, 0.50; MgSO_4_, 0.20; and CaCl_2_·2H_2_O, 0.04. All nutrients were dissolved in redistilled water containing (per liter) 6 mL of metal solution (750 mg Na_2_EDTA, 97 mg FeCl_3_·6H_2_O, 41 mg MnCl·4H_2_O, 5.0 mg ZnCl_2_, 2 mg CoCl·6H_2_O, and 4.0 mg Na_2_MoO_4_·2H_2_O) and 1 mL of trace-nutrient solution (50.0 mg Na_2_EDTA, 618 mg H_3_BO_3_·6H_2_O324.7H, 19.6 mg CuSO_4_·5H_2_O, 44.0 mg ZnSO_4_·7H_2_O, 0.20 mg CoCl_2_·6H_2_O, 12.6 mg MnCl_2_·4H_2_O, and 12.6 mg Na_2_MoO_4_·2H_2_O).

### 2.4. Cultures Treatments

Nitrogen was supplied as NaNO_3_ with the concentrations of 1.25, 2.5 g/L, and 5.0 g/L NaNO_3_ into a different flask containing 1.7-liter Zarrouk medium in a 2 L flask. Aeration was accomplished utilizing air pumps to achieve an air flow rate of 20 L/h. The cultures were gassed with 0.03% volume CO_2_ in the air and temperature was maintained at 25°C ± 3°C. The pH of all media was adjusted to 9.5 and monitored at 24 h intervals. The cultures were illuminated with perpetual 10 cool white fluorescent lamps (Philips 40 W) that provided an illumination of 2500 lux. In all cultivated flasks, conductivity, salinity, pH, and temperature were daily quantified with Hanna (HI 09812-5) conductivity meter. The purity of cultures was periodically checked by microscopic observation following taxonomy guidelines. All solutions and glassware were autoclaved at 121°C for 15 min prior to utilization.

### 2.5. Cultivation of* S. maxima* at Large Scale for Production of L-Asparaginase

The inoculum from 5 g/L NaNO_3_ *Spirulina maxima* culture was cultured in 320 L glasses aquarium containing 300 L Zarrouk medium. The aquarium was incubated at the same conditions as described previously.

### 2.6. Growth Measurements

The growth rate of* S. maxima* was monitored every three days through the entire cultivation period by determining the dry weight (dw) and optical density (OD) at 560 nm by UV-vis spectrophotometer [18]. A good linear relationship between the biomasses DW concentration and the OD 560 nm was recorded. All analytical determinations were performed in triplicate and the mean values were recorded.

### 2.7. Harvesting

The algal cells were harvested at the stationary phase, by centrifugation at 10,000 ×g (4°C) for 15 min, and the cell masses were stored at −20°C until analysis.

### 2.8. Rapid Screening of L-Asparaginase Production by Phenol Red Assay

Rapid screening of L-asparaginase produced from SM cultures was assessed based on Gulati et al. [19] method with the incorporation of pH indicator phenol red (prepared in ethanol) in MS L-asparagine. Phenol red at acidic pH is yellow and at alkaline pH turns pink; thus a pink color is formed by algal cultures producing L-asparaginase. Screening of potential L-asparaginase producing algae was carried out with the use of asparagine; pH was adjusted to 6.8 and supplemented with phenol red as a pH indicator (0.009% final concentration). Tubes were examined for change in color of cultures from yellowish to pink due to change in pH indicating the positive asparaginase activity and used for further study.

### 2.9. Extraction of Crude L-Asparaginase Enzyme

Extraction of crude enzyme was done by adding 10 mL of sodium phosphate buffer (pH 7) to the MS cultures, kept in rotator shaker for 45 min. One mL of the extract was transferred to the Eppendorf tube and centrifuged at 10,000 ×g for 10 minutes. The obtained supernatant was used as crude extract for L-asparaginase assay.

### 2.10. L-Asparaginase Enzyme Extraction and Assay

L-asparaginase activity was determined by measuring the amount of ammonia released by nesslerization according to Wriston Jr. and Yellin [20] method. In brief, 0.2 mL of cell-free supernatant was mixed with 0.8 mL of 0.1 M sodium borate buffer (pH 8.5) and 1 mL of 0.04 M L-asparagine and the reaction mixture was incubated for 10 min. Then, 0.5 mL of 15% TCA was added to stop the reaction and centrifuged at 10000 ×g for 10 minutes. 0.2 mL of the supernatant was taken in a test tube and 3.6 mL distilled water was added followed by additional 0.2 mL Nessler's reagent. The absorbance was measured spectrophotometrically at 480 nm. The enzyme activity was expressed in IU. One IU of L-asparaginase is the amount of enzyme which liberates 1 *μ*mole of ammonia per mL per min (*μ*mole/mL/min) at 37°C.

### 2.11. Purification of L-Asparaginase

The purification was carried out using crude enzyme extract. The enzyme was purified by the following steps: ammonium sulfate precipitation and Sephadex G-200 gel filtration, according to the modified method of Distasio et al. [21, 22]. After each step, the L-asparaginase activity and total protein content were determined.

### 2.12. Ammonium Sulfate Fractionation

The powder of ammonium sulfate was added gradually to the crude extract (20–80%) reaching 80% saturation solution. The mixture was left for 12 h at 4°C, followed by centrifugation at 8,000 ×g for 20 min at 4°C. The fractions precipitate was dissolved in a 0.01 M phosphate buffer pH 8.5 and dialyzed overnight against the same buffer at 4°C.

### 2.13. Sephadex G-200 Gel Filtration

The dialyzed ammonium sulfate fraction was applied to a Sephadex G-200 column that was preequilibrated with a 0.01 M phosphate buffer pH 8.5. Then, the protein fraction was eluted with the 0.01 M phosphate buffer at a flow rate of 5 mL/25 min. It was assayed for protein quantity at 280 nm as well as for enzyme activity. The active fractions were pooled, dialyzed against the 0.01 M phosphate buffer pH 8.5, and concentrated.

### 2.14. Determination of Soluble Protein Content

The total protein content in soluble protein content and protein fractions was determined spectrophotometrically at 595 nm, using Coomassie Blue (G 250) as mentioned by Bradford [23]. Bovine serum albumin (BSA) was used as a protein standard to preparation of calibration curve.

### 2.15. Kinetic Properties of L-Asparaginase Isolation from* S. maxima*

To determine the optimum pH and temperature of the AsnA enzyme, the activity of the purified* S. maxima* L-asparaginase was assessed by incubating the assay mixture under assay conditions as a function of varying ranging of pH (pH 4.0, 7.0, 8.0, and 9.5) and temperature (20–50°C).

### 2.16. Optimum of pH

The optimum pH of the enzyme was determined as reported by Singh et al. [24] using the following buffers: at 0.05 M concentration: sodium acetate buffer (pH 5-6), potassium phosphate buffer (pH 6.5–7.5), and Tris-HCl buffer (pH 8.0–9.5). Blank assays were carried out without adding enzymes. The pH degree studies were carried out by preincubating the enzyme (AsnA) at different pH for 15 min and then the residual activity was measured.

### 2.17. Optimal Temperature

The optimal temperature of purified L-asparaginase enzyme activity was determined by preincubating the enzyme at desired temperature (20–50°C) for 15 min and the amount of ammonia liberated was determined. At each temperature, blank assay was done without adding the enzyme.

### 2.18. Statistical Analysis

All measurements were carried out in triplicate. Statistical analyses were performed using one-way analysis of variance (ANOVA), and the significance of the difference between means was determined by Duncan's multiple range tests. Differences at *P* < 0.05 were considered statistically significant. The results were presented as mean values (±SD, standard deviations).

## 3. Results and Dissection

### 3.1. Effect of Nitrogen Concentration on Growth of* Spirulina maxima*

**Figure 1:**
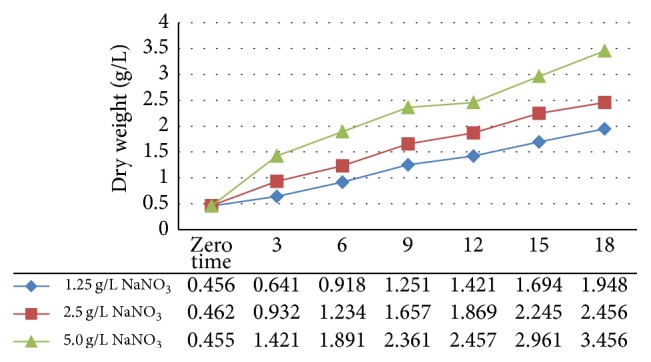
Effect of nitrogen concentration on growth of* Spirulina maxima*.

Nitrogen is the major structural and functional element of algal cells and plays an important role in the nutrition in states. Nitrogen is required for all biosynthesis leading to reproduction, product formation, and cell maintenance. Production of the primary (proteins and carbohydrates) metabolite by microorganisms is highly influenced by their growth conditions.  Table 1 and Figure 1 show the effect of N_2_ (as NaNO_3_) concentrations (1.25, 2.50, and 5.0 g/L) in Zarrouk medium on algae growth for 18 days, as monitors by dry weight yields of biomasses (DWYg/L). The results showed that the DWY was significantly different (*P* > 0.5%) among all algae cultures at the incubation time interval. The biomass yield (g/L DW) was increased significantly among all cultures as the function of incubation times and nitrogen concentrations. The highest biomass yield recorded after 18 days of incubation was 3.456 mg/L, 2.456 mg/L, and 1.948 mg/L in SM cultures grown in Zarrouk's medium containing 5.0 g/L, 2.5 g/L, and 1.25 g/L N_2_, respectively. Thus, high biomass yield was noticed in* S. maxima* cultured in either high (5.0 g/L) or optimal (5.0 g/L) N_2_ concentrations compared with that noted in low N_2_ (1.25 g/L) culture. It is known well that nitrogen is an essential element of* S. maxima* growth and it grows well with high biomass yield [25]. Earlier studies also similarly showed that the yield of* A. platensis* biomass grown in rich or optimal N_2_ was higher than that in low N_2_ cultures [26]. However, it is important to note that certain algae species have the ability to adapt to the nutrient limitation (such as nitrogen, iron, and phosphorous) and stress environmental conditions (such as high light intensity and high salinity) by changes in its metabolic pathway [26, 27]. Similar results were reported in the literature [25, 28] that some algae species had great abilities to induce biomolecules including lipid, protein, and carbohydrates biosynthesis when grown in medium containing high N_2_ concentration [26]. In this context, our previous study showed that the created microalgae species grown under salt stress coupled with high N_2_ levels had high protein content. In general, environmental factors, nutrient status, and salinity lead to changes in cellular metabolic pathway and cellular composition [26]. These changes may be achieved at the expense of other main components such as lipids and carbohydrates.

### 3.2. Effect of Nitrogen Concentration on Protein Constituents of* Spirulina maxima*

Data in Table 2 shows the effect of nitrogen concentration on protein parameters including total protein (% of dry weight (DW), TP), soluble protein (mg/g, SPr), and soluble protein yield (mg/L, SPY) concentrations of* Spirulina maxima* (SM) growth in Zarrouk medium. These results indicate that the increase of nitrogen concentration in the Zarrouk nutrient media led to high changes in TP, SPr, and SPY contents in SP cells. At high N_2_ 5.0 g/L, the values of these constituents were 56.36% of DW, 40.52 mg/g of DW, and 1397.9 mg/L, respectively. At limited N_2_ 1.225 g/L, these values were 33.42% of DW, 20.12 mg/g of DW, and 390 mg/L, respectively. Thus, the protein constituents were increased in algal cells grown at high N_2_ by 2.51%, 4.11%, and 9.01%, respectively, as great as that in SP grown at low N_2_ concentration. Thus, the increase of N_2_ concentration in nutrient medium caused a significant increase of protein constituents in SM cultures. It could be noted that the total protein content increased with the increasing of N_2_ concentration in Zarrouk media. By this way, increasing the concentration of N_2_ in the nutrient medium over that optimal level can be manipulated with respect to its total proteins and soluble protein content. One would say that increase in N_2_ levels in growth medium may lead to increase in the protein synthesis required for increasing the metabolic pathway intracellular nitrogenous compounds in order to balance the high N_2_ concentration. However, it is well known that the nitrogen concentration in a medium has a great influence on protein content and its constituents in several species of* Spirulina* species. Piorreck et al. [27] reported that* Spirulina*,* Chlorella*, and many algae species grown in high nitrogen level showed high protein contents. Zeng and Vonshak [29] found that* Spirulina* cells grown under stress conditions, including salinity-stress, have a lower protein synthesis capacity. The finding results are in harmony with that of our findings [26] in* Spirulina* grown in medium containing high nitrogen with a high biomass production and total protein content as factors for macro- and microelements such as nitrogen source and concentrations. On the other hand, Becker [30] and Abd El-Baky and El-Baroty [26] reported that* Spirulina* spp. grown in a nitrogen-rich medium had a high ability to accumulate a considerable quantity of proteins (>60%) and up to 20% of this protein fraction was identified as a phycocyanin blue pigment.

### 3.3. Repaid Screening of L-Asparaginase Production by Tube Assay

**Figure 2:**
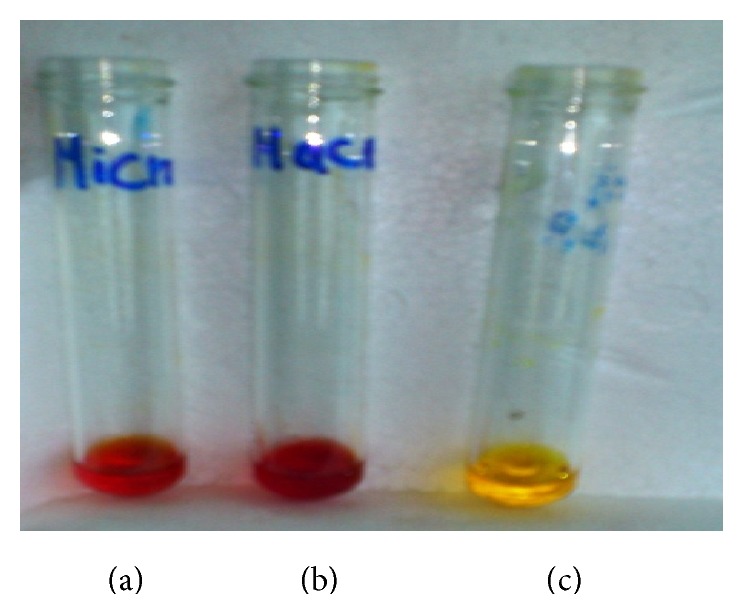
Detection of L-asparaginase (L-AsnA) activity of macroalgae and microalgae assayed by Gulati et al. [19] method using phenol red indicator: (a) L-AsnA production from microalgae, (b) L-ASase production from macroalgae, and (c) control without L-AsnA extract.

L-asparaginase (L-AsnA) activity of microalgae was rapid assay based on Gulati et al. [19] method. L-asparaginase production in* Spirulina maxima* grown in Zarrouk medium that contained various nitrogen concentration was colorimetrically assessed based on pH indicator phenol red (PR). Algal L-asparagine was incorporated into 0.009% methanolic phenol red-pH indicator; phenol red at acidic pH is yellow and in alkaline pH turns into pink colors. Thus, appearing of pink color indicates the hydrolysis of L-asparagine into aspartic acid and ammonia was released by L-asparaginase (L-AsnA) presence in* Spirulina maxima* (SM). Here, again, initial pH was changed from acidic (yellow) to basic (pink) due to the release of ammonia and this could be considered as positive result. After 5 min incubation period, aliquot* Spirulina* extract with PR was changed to pink coloration and it indicates the presence of algal L-asparaginase with high activity (Figure 2). However, the depth of color intensity was increased with increasing L-AsnA activity and the deep pink colors were considered as L-AsnA producing species. Any algae cultures exhibiting L-AsnA activity were chosen for further study.

### 3.4. Effect of Nitrogen Concentrations on Enzyme Activity of L-Asparaginase Produced by* Spirulina maxima*

The enzyme activity of asparaginase produced by* S. maxima* grown in various nitrogen cultures was determined by evaluating enzyme activity IU, total protein, specific activity, and yield of enzyme in an aliquot of the SM enzyme extract (Table 3). In rich N_2_ algae cultures, these values were 898 IU, 405 mg/g, 2.21 IU/mg protein, and 51.28 IU/L, respectively. At low N_2_ and optimal N_2_ (in parenthesis), these values were found to be 356 IU (594 IU/L), 212 mg/g (332 mg/g), 1.667 IU/mg protein (1.79 IU/mg protein), and 32.39 IU/L (43.86 IU/L), respectively. This result revealed that the highest enzyme activities were recorded in SM cultures grown in high N_2_ medium when compared with that in optimal and low N_2_ cultures. Thus, algae grown in rich N_2_ medium were considered as high substrates and high enzyme activity correlated with good algae biomass yield. This result also showed significant differences among all various nitrogen cultures on the production of asparaginase by* S. maxima*.

### 3.5. Purification and Characterization of L-Asparaginase

Among all three SP cultures examined for synthesis of L-asparaginase, SP culture grown in high N concentration medium characterized by high yields enzyme 51.28 U/L and high enzyme activity (898 IU) and protein enzyme (405 mg/g) content was selected for further studies. The characterization parameters of its high synthesis of L-asparaginase in* S. maxima* cultures purification of crude enzyme was performed by 80% ammonium sulfate precipitation and gel filtration GF purification steps is summarized in Table 4. Both the purification methods showed a significant effect to improve the enzyme characterization as high as that in crude enzyme extracts. The values of enzyme activity IU, total protein mg/g in aliquot of enzyme extract, specific activity IU/mg protein, and yield of enzyme IU/L (Table 2) of 80% AS and GF (in parenthesis) purification methods were 515 IU (485 IU), 485 mg/g, 55.4 IU/mg protein (25.4 IU/mg protein), and 9.24 IU/L (19.1 IU/L), respectively. These values revealed that the AsnA enzyme of* S. maxima* purified by GF had a high specific activity of 19.1 IU/g and was found to be of approximately 2-fold purity compared to 80% AS purification method. However, 80% AS and GF purified process showed a high enzyme purification 5.28- and 10.91-fold greater than that in crude enzyme extract (Table 3). However, the percentages of the enzyme yield (%) of purified L-asparaginase crude extract by 80% AS precipitation and GF were 91.26% and 86.45%, respectively, of crude extract (%). Thus, the enzyme yield was retained after purification steps with 91.26% and 86.45 of crude enzyme. Thus, in this study, L-asparaginase was precipitated at 80% ammonium sulfate which leads to high fold in yield and purification of enzyme but it was less than that by GF. The total soluble protein content was decreased from 321.2 mg/mL of its original concentration of 55.4 and 25.4 mg/mL in purified AsnA enzyme by using AS and GF purification steps, respectively (Table 3). El-Bessoumy et al. [5] found that the purified L-AsnA from* Pseudomonas aeruginosa* reached 106-fold by 3 steps of purification (AS precipitation and GF on Sephadex G-100 column followed by CM-Sephadex C50 column).

### 3.6. Physiochemical Properties of L-Asparaginase from SM Culture

**Figure 3:**
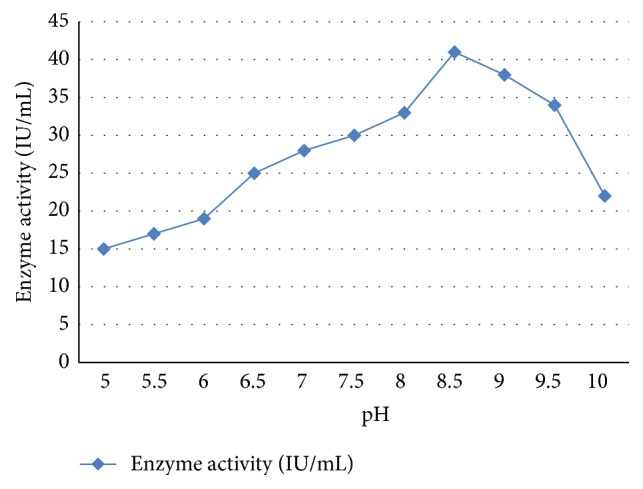
Effect of pH on* Spirulina maxima* L-asparaginase activity. Enzyme samples (containing 1 mg/mL of protein) were incubated at pH ranges from 5 to 10.

**Figure 4:**
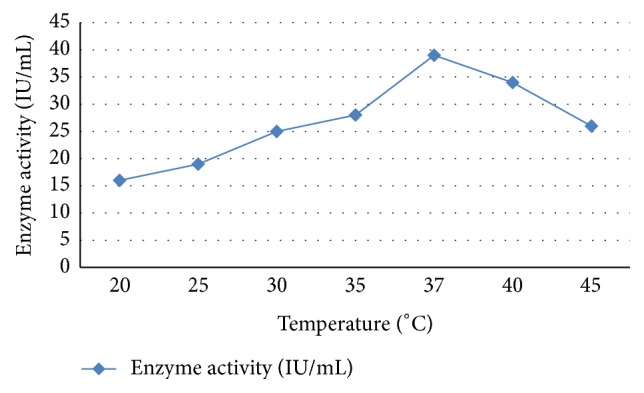
The effect of temperature on the stability of L-asparaginase activity. Enzyme samples (containing 1 mg/mL of protein) were incubated at temperature ranges from 20 to 45°C.

In the final purification steps, GF enzyme showed a high specific activity of 19.1 IU/mg, with approximately 12-fold purity and 68.45 enzyme yields. Therefore, the optimal pH and the temperature degrees of* S. maxima* AsnA enzyme purified by GF step are (Figures 3 and 4) determined. The results showed that the enzyme exhibited maximum activity 42 IU/mL at pH 8.3 and 8.5 and optimum temperature was 36.5°C–37°C. Similar results were found in many microbial species such as* Mycobacterium* spp.,* S. ginsengisoli*, and* Pseudomonas* spp. and optimum temperature was 37°C and optimum pH was in the range of 8–8.5, which is close to optimum pH recorded for L-asparaginase obtained in this study [9, 31].

In general, the optimum activity of purified AsnA enzyme was recorded at the pH 8.5 and 37 ± 0.42°C the optimum temperature in many of microbial species. In this regard, El-Bessoumy et al. [5] reported that maximum activity of L-asparaginase of* P. aeruginosa* was obtained at pH 8.5 and optimal temperature 37 ± 0.42°C. The enzyme from* Enterobacter cloacae* had a pH and temperature optimum of 8.5 and 37 ± 42°C, respectively [9]. In contrast, the asparaginase from* P. geniculata*,* P. stutzeri*, and* Aspergillus niger* has a pH optimum of 9.0 [8]. The physicochemical properties of L-asparaginase from* S. maxima* are within the range reported for the L-asparaginases of many microorganisms, in the alkaline region (pH 8-9). Makky et al. [31] reported that the specific activity of the pure enzyme isolated by* Streptobacillus* sp. was recorded to be 21.77 U/mg with 39.58-fold purification and 39% of yield. Finally, the maximum enzyme activity of* S. maxima* was found to be at/or near physiological pH and temperature, making it extremely valuable in the chemotherapeutic treatment of some diseases such as cancer.

## 4. Conclusion

*Spirulina maxima* (SM) cultures grown in Zarrouk medium containing high N_2_ (5 g/L) as NaNO_3_ level for 18 days showed high dry biomass yields (1.948 g/L) and L-asparaginase (L-AsnA) enzyme production, total protein 405 mg/g, specific activity 2.21 IU/mg protein, and yield of enzyme 51.28 IU/L compared with those obtained in either optimal N_2_ (2.5 g/L) or low N_2_ (1.25 g/L) concentration culture. The partial purification of crude MS L-asparaginase enzyme achieved by 80% ammonium sulfate prepetition and gel filtration had a high specific activity greater by 5.28 and 10.91 than that in crude enzyme extract. The optimum activity of purified enzyme was recorded at 8.5 and 37 ± 0.2.

## Figures and Tables

**Table 1:** Effect of nitrogen concentration on growth of* Spirulina maxima.*

Nitrogen concentrations as NaNO_3_	Biomass (dry weight g/L)							LSD^a^
	Zero time	3	6	9	12	15	18	
1.25 g/L	0.456	0.641	0.918	1.251	1.421	1.694	1.948	0.11
2.5 g/L control	0.462	0.932	1.234	1.657	1.869	2.245	2.456	0.16
5.0 g/L	0.455	1.421	1.891	2.361	2.457	2.961	3.456	0.21
LSD^b^	0.13	0.14	0.12	0.15	0.23	0.34	0.36	

Mean values of three replicates, LSD.

a: Least Significant Difference between any two means of time. b: Least Significant Difference between any two means of concentration.

**Table 2:** Characterization of* Spirulina maxima* protein grown at nitrogen concentrations.

Nitrogen concentrations as NaNO_3_	Total protein % DW	Soluble protein % WD	Soluble protein yield mg/L
1.25 g/L	33.42 ± 1.12	20.12 ± 1.22	390 ± 5.44
2.5 g/L control	42.34 ± 1.16	33.23 ± 1.65	814 ± 16.45
5.0 g/L	56.36 ± 2.32	40.52 ± 2.11	1397.9 ± 51.33

Mean values of three replicates, ±SD.

**Table 3:** Effect of nitrogen concentrations on L-asparaginase production by* Spirulina maxima.*

Nitrogen concentrations as NaNO_3_	Enzyme activity	Protein	Specific activity	Yield of enzyme
	IU	mg/g	IU/mg	IU/L
1.25 g/L	356 ± 6.2	212 ± 3.5	1.67 ± 0.21	32.39 ± 2.6
2.5 g/L control	594 ± 6.5	332 ± 5.2	1.79 ± 0.11	43.86 ± 2.5
5.0 g/L	898 ± 11.6	405 ± 6.5	2.21 ± 0.22	51.28 ± 3.4

Mean values of three replicates, ±SD.

**Table 4:** Purification of L-asparaginase from *Spirulina maxima.*

Sample	Enzyme activity IU	Protein mg	Specific activity IU/g	Fold purification	Enzyme yield%
Crude	561	321.2	1.75	0.0	100
Ammonium sulfate 80%	512	55.4	9.24	5.28	91.26
Gel filtration	485	25.4	19.1	10.91	86.45
